# Toe Acrometastasis as an Initial Sign of Metastasis in a Patient With a Large Cell Lung Carcinoma

**DOI:** 10.7759/cureus.30861

**Published:** 2022-10-30

**Authors:** Zakhia Michael Malhame, Rami El Rachkidi

**Affiliations:** 1 Department of Orthopaedics and Traumatology, Notre Dame des Secours University Hospital Medical Center, Byblos, LBN; 2 Department of Orthopaedics and Traumatology, Bhannes Medical Center, Bhannes, LBN; 3 Department of Orthopaedics and Traumatology, Hôtel-Dieu de France Hospital, Beirut, LBN

**Keywords:** pain, palliative management, foot, toe, lung carcinoma, acrometastasis

## Abstract

Acrometastases are rare lesions that originate most commonly from the primary lung cancer. They can mislead the diagnosis and the treatment, since they often appear as an osteomyelitis of the affected area. The presence of these metastases is a sign of poor prognosis, with a life expectancy of few months. We report a case of a 78-year-old male with an acrometastasis to the distal phalanx of the right fourth toe. It was the first sign that his previously diagnosed large cell lung carcinoma had reached a metastatic stage. Amputation of the toe was considered in this case where the intense pain of his acrometastasis could not be managed even with strong analgesics.

## Introduction

Metastases to the bone are a common site of secondary localization of certain types of cancer. However, acrometastases, defined as metastases below the elbow or the knee, occur in only 0.1% [1]. In the case of lung carcinoma, the literature shows a higher rate of metastasis to the hands compared to the foot. The latter is exceptional especially in the case of forefoot [2]. These acrometastases are usually a sign of a terminal stage cancer, thus divulgating a bad prognosis with a mean life expectancy of six months. Acrometastases are most commonly found in patients who have a tumor in the lungs, and less commonly in other types of cancers [3,4]. Here, we report the case of a 78-year-old man with a large cell carcinoma metastatic lesion to the distal phalanx of a toe. He suffered from excruciating pain in his foot that dramatically affected his daily activities, and needed a radical solution.

## Case presentation

**Figure 1:**
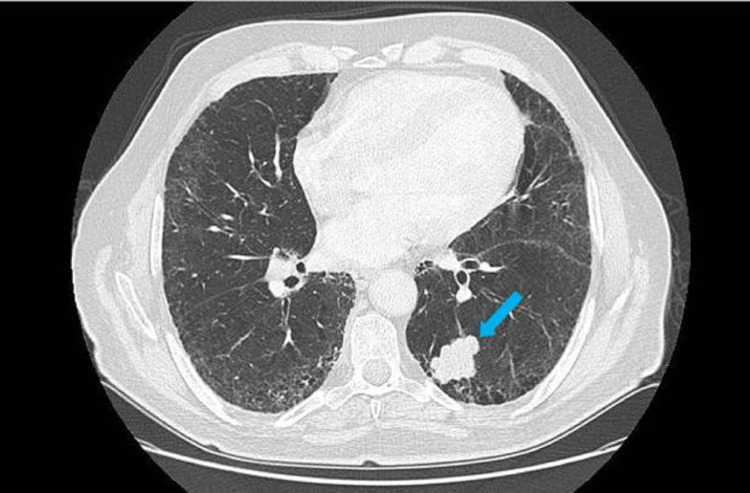
A mass (blue arrow) seen in the left posterior-basilar lung on chest CT .

A 78-year-old Caucasian male, with a history of 40 pack-year cigarette smoking and percutaneous transluminal coronary angioplasty (PTCA) done 15 years ago, was referred to our orthopedic department for an acrometastasis of his distal phalanx of the fourth toe of the right foot. His report showed that he presented to the emergency department, four months ago, with shortness of breath. He was diagnosed, based on a chest radiograph, with bilateral pulmonary edema and fluid overload. Echocardiography showed a very low ejection fraction of 25%. He was treated symptomatically with furosemide and discharged few days later on furosemide, Entresto, spironolactone and Procoralan. Two weeks after, he was rushed again to the emergency department for hypotension and dyspnea. This time, a chest computed tomography (CT) scan was performed, and a mass was detected in the left posterior-basilar lung along with fibrotic changes in the lung parenchyma (Figure 1).

A biopsy of the mass was accomplished, and he was diagnosed with a poorly differentiated large cell carcinoma of the lung (Table 1).

**Table 1:** Immunostaining and immunohistochemistry properties of the tumor TTF-1, thyroid transcription factor 1; TPS, tumor proportion score

	Results
Immunostaining	Cytokeratin 7 positive
	Cytokeratin 5/6 negative
	TTF-1 negative
	Ki67 >70% of the tumor cells positive
	Synaptophysin negative
	Chromogranin negative
	CD-56 very few cells positive
	p63 exceptional tumor cells positive
	Napsin A negative
Immunohistochemistry staining for PD-L1	Percentage of positive cells (TPS): 0%

The positron emission tomography (PET) scan showed a single tumor of the left lower lobe without adenopathies, or extrathoracic disease, with changes suggestive of pulmonary fibrosis. The brain MRI did not show any particular changes. His blood test results were insignificant.

**Figure 2:**
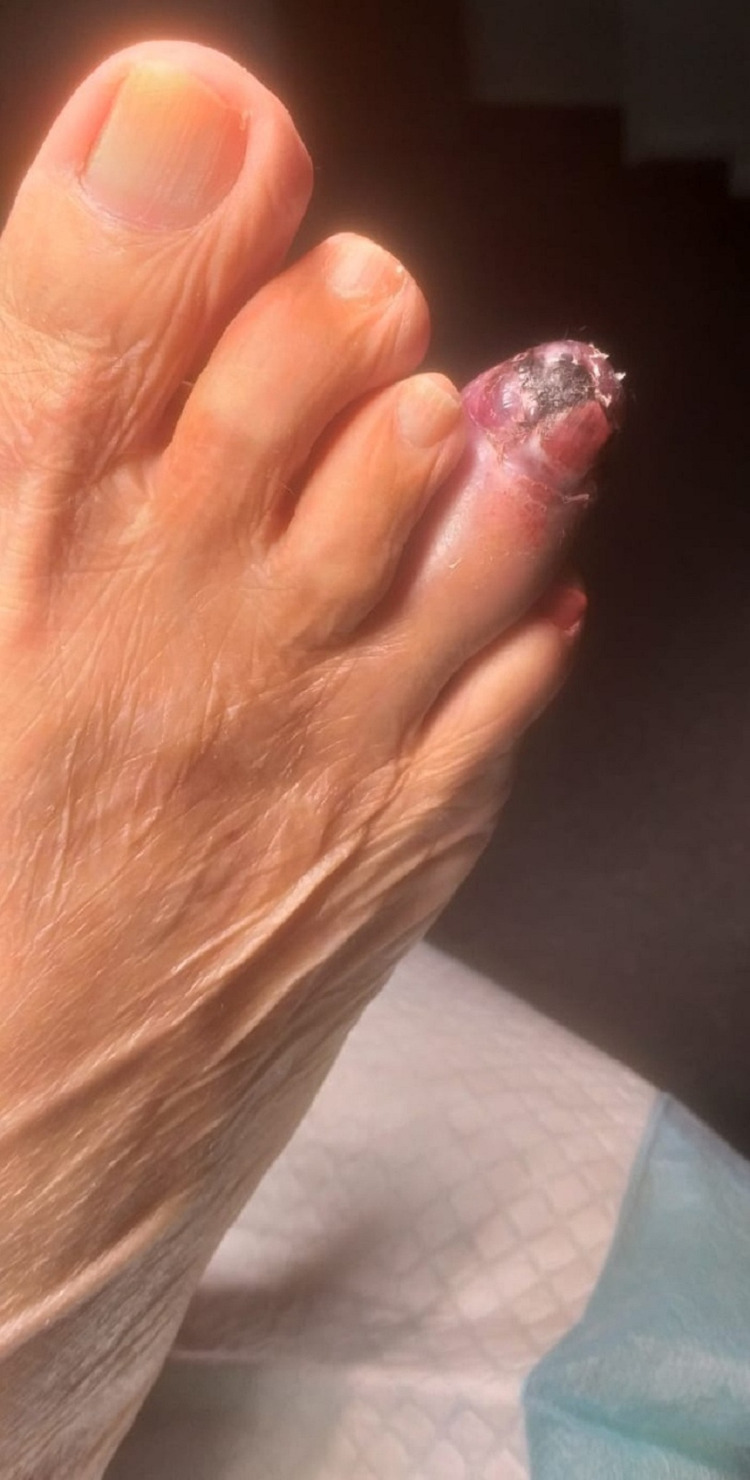
Dorsal view of the toe before amputation

**Figure 3:**
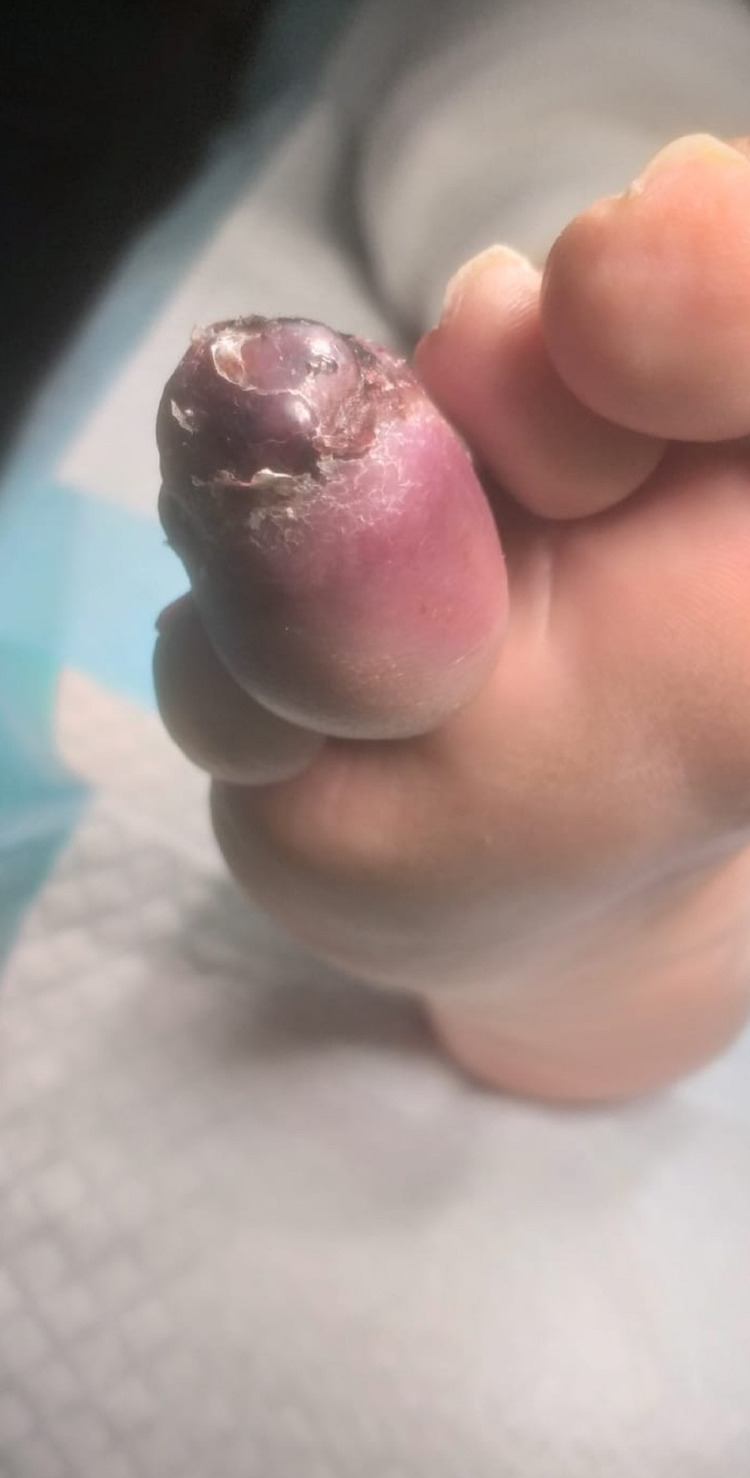
Plantar view of the toe before amputation

His case was discussed in a multidisciplinary meeting between the oncology team, anesthesiologists, and cardio-thoracic surgeons. Due to the cardiac insufficiency and the pulmonary fibrosis, it was decided that the patient would not tolerate chemotherapy or a lobectomy. Instead, he was referred for radiation to the chest after his consent. The patient underwent five sessions of radiotherapy at a dose of 4500 cGy in 11 days. After the completion of the radiation sessions, the patient had a WHO performance score of 0 and was asked to perform a total body CT scan for follow-up after few weeks. A month later, the patient started feeling a burning type of pain and noticed some swelling in the fourth toe of his right foot, without a history of trauma or diabetes. His doctor primarily suspected a soft tissue infection, and he prescribed an amoxicillin/clavulanate regimen for seven days. After completion of the antibiotic treatment, the swelling, erythema and pain were still increasing (Figures 2, 3).

**Figure 4:**
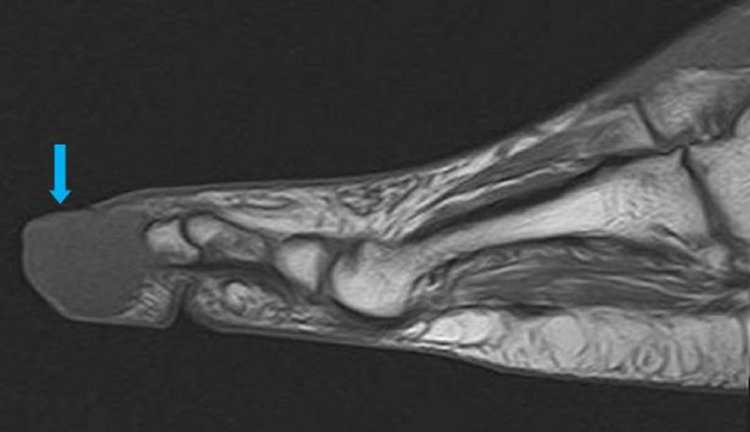
Sagittal view on the MRI T1 sequence visualizing the mass on the distal phalange of the fourth toe (blue arrow)

**Figure 5:**
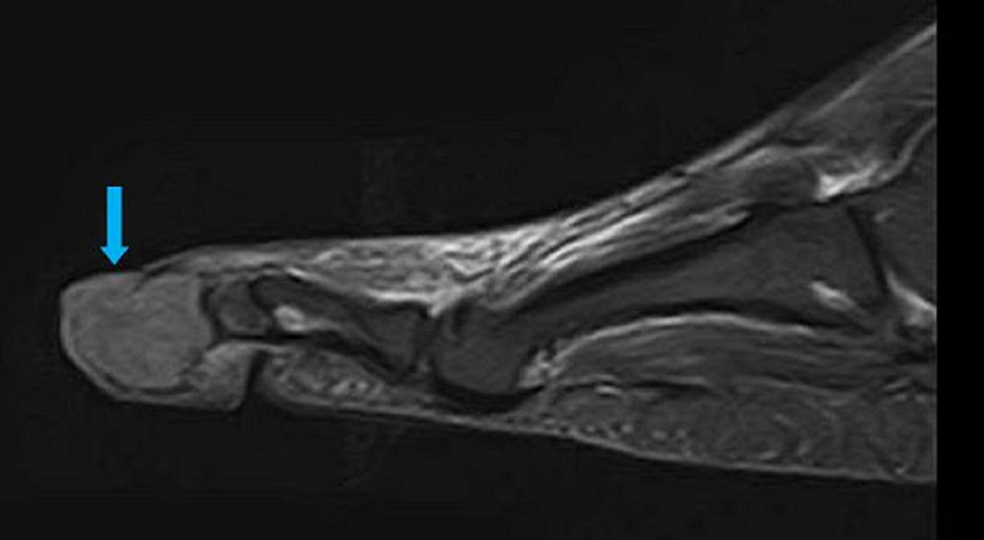
Sagittal view on the MRI T2 STIR sequence visualizing the mass on the distal phalange of the fourth toe (blue arrow) STIR, short tau inversion recovery

**Figure 6:**
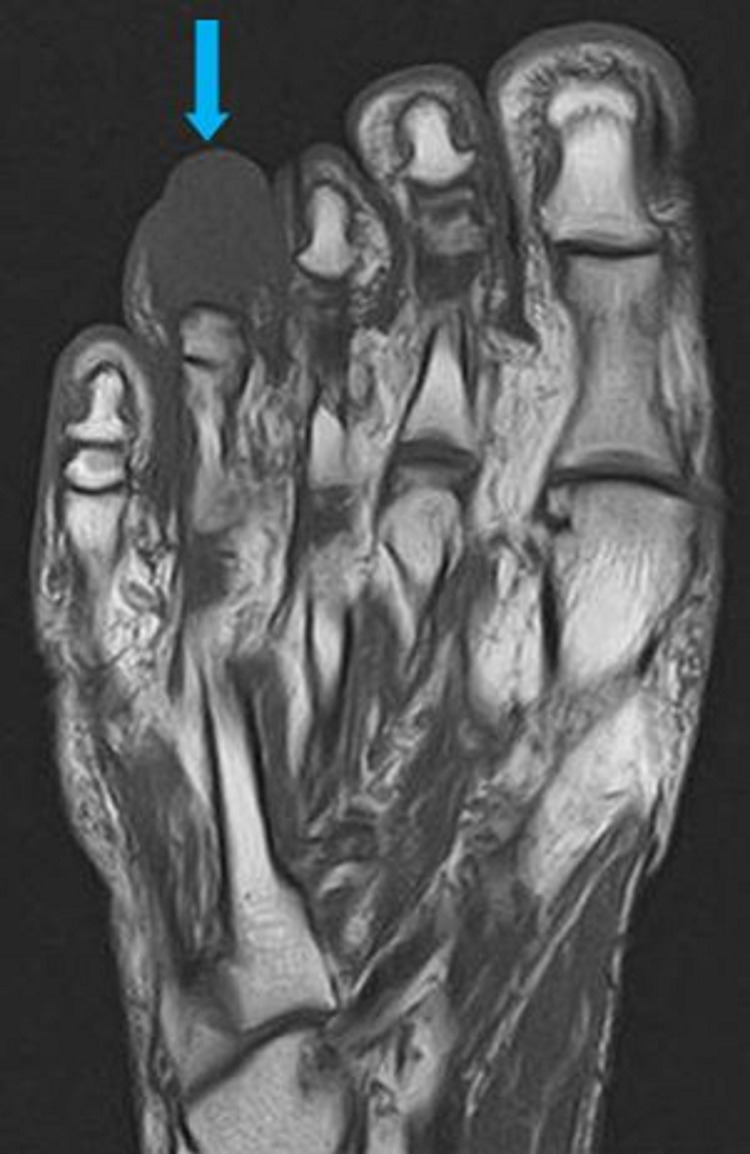
Coronal view on the MRI T1 sequence visualizing the mass on the distal phalange of the fourth toe (blue arrow)

**Figure 7:**
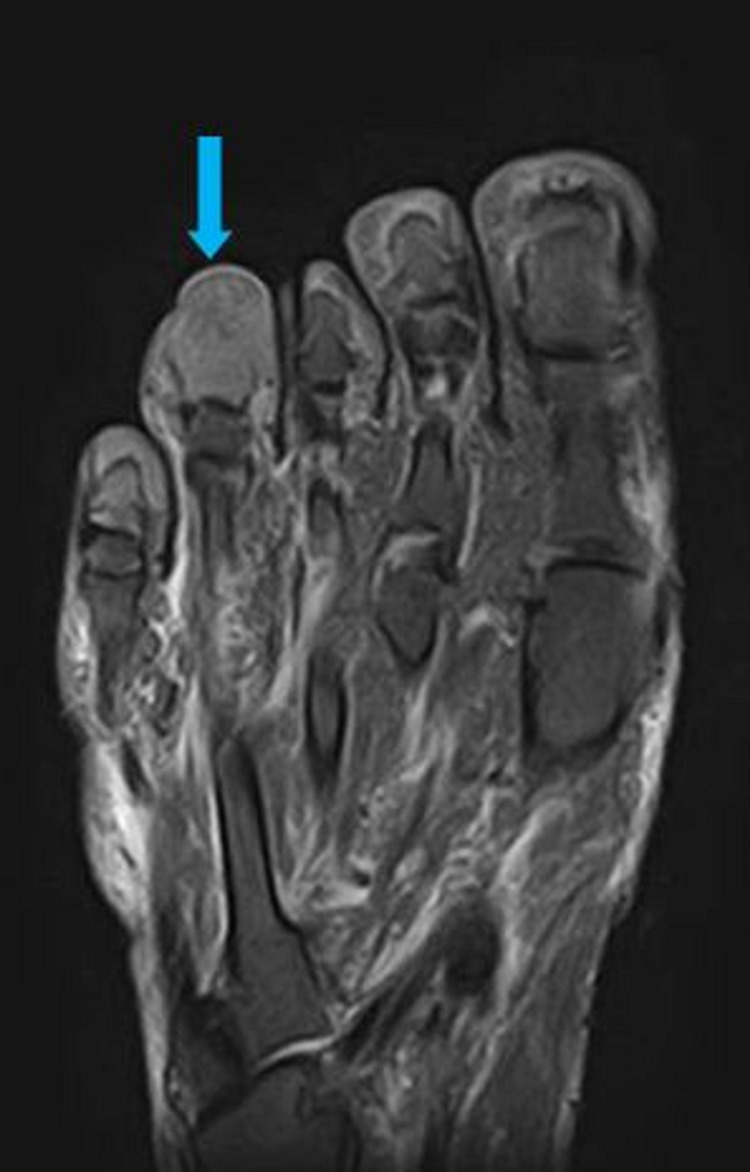
Coronal view on the MRI, T2 STIR sequence visualizing the mass on the distal phalange of the fourth toe (blue arrow) STIR, short tau inversion recovery

An MRI of his right foot was performed to rule out an osteomyelitis. A soft-tissue mass was seen on the MRI in the fourth toe measuring 20 x 17 mm, hyposignal with T1 and weak hypersignal with T2 short tau inversion recovery (STIR), leading to a total osteolysis of the distal phalanx (Figures 4-7).

**Figure 8:**
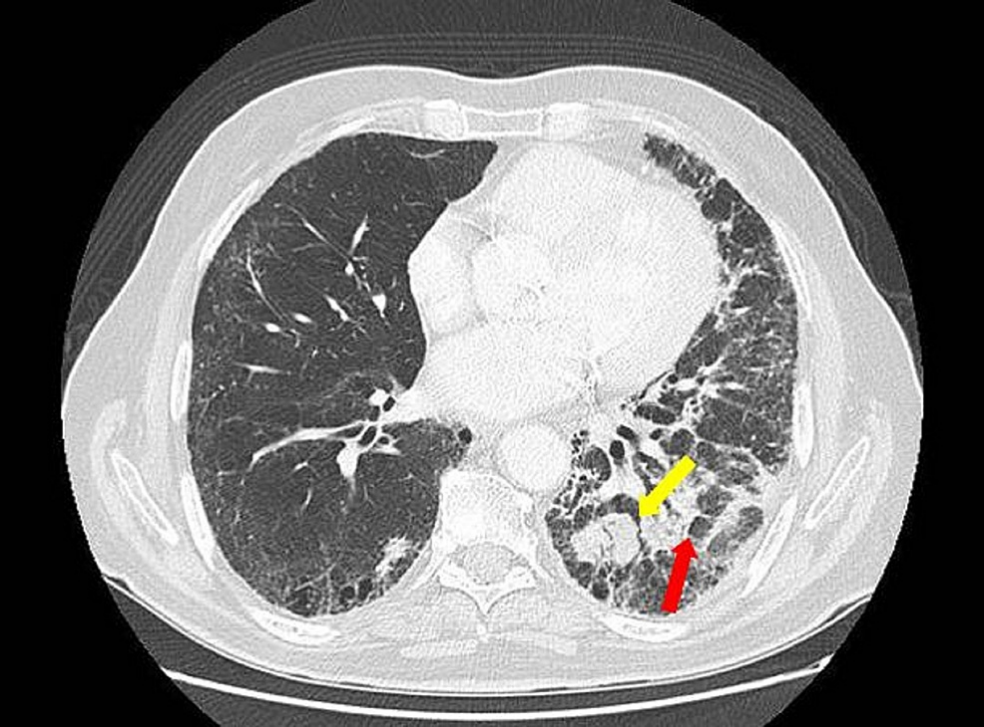
CT showing regression of the pulmonary tumor, presence of multiple bilateral mediastinal and hilar adenopathies, as well as fibrotic changes in the left posterior-basilar lung The yellow arrow is showing the mass; the red arrow is showing fibrosis of the parenchyma.

The aspect of the mass was highly suspicious of a secondary localization of the large cell carcinoma, given the patient history. No other anomaly of the toe or the foot was seen. On the light of this discovery, he underwent a total body CT scan, which had been already planned but not completed a month earlier. The chest CT, compared to the imaging done before radiotherapy, showed 30% regression of the size of the pulmonary tumor, the appearance of multiple bilateral mediastinal and hilar adenopathies, as well as fibrotic changes of the left posterior-basilar lung, most probably due to radiation (Figure 8). On the abdominal level, multiple metastases were seen only in the liver, without adenopathies.

**Figure 9:**
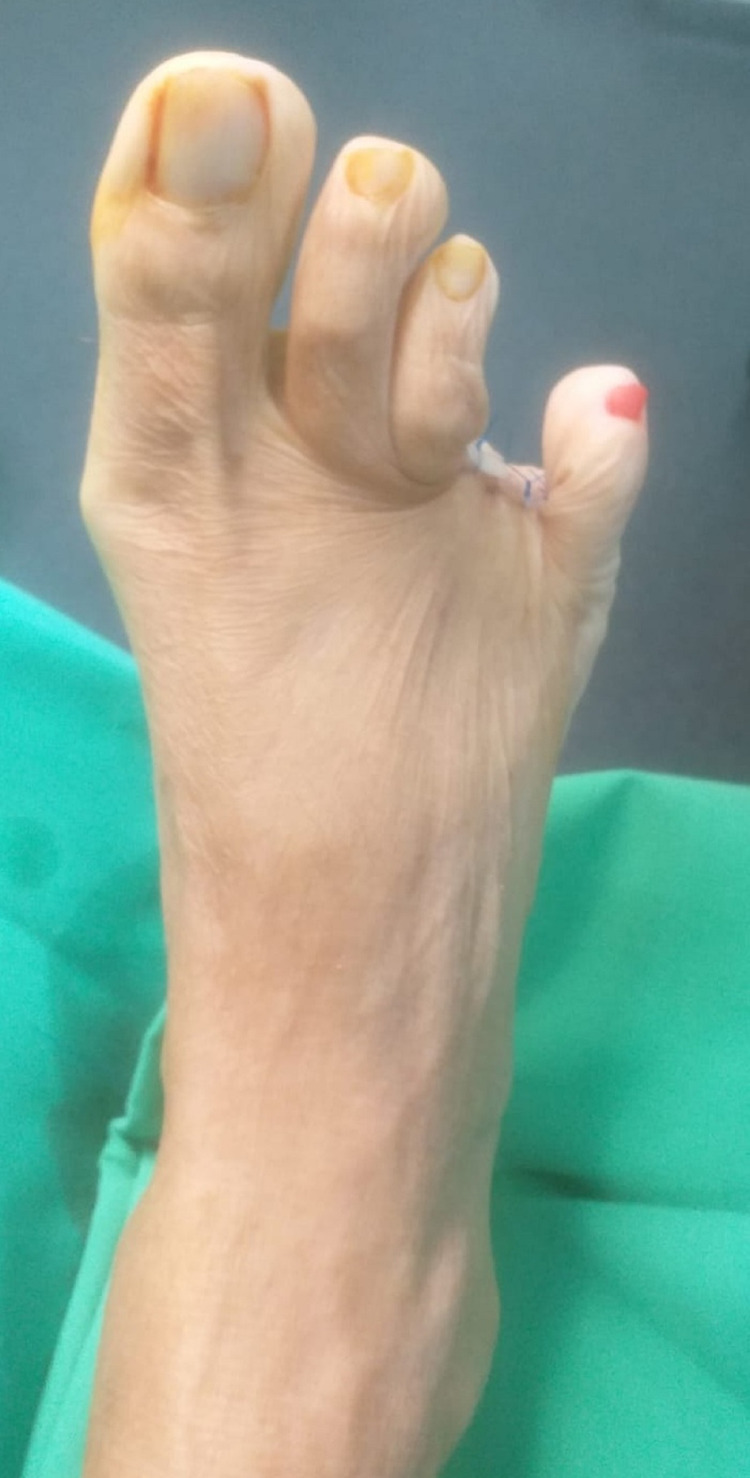
Dorsal view of the foot after amputation

**Figure 10:**
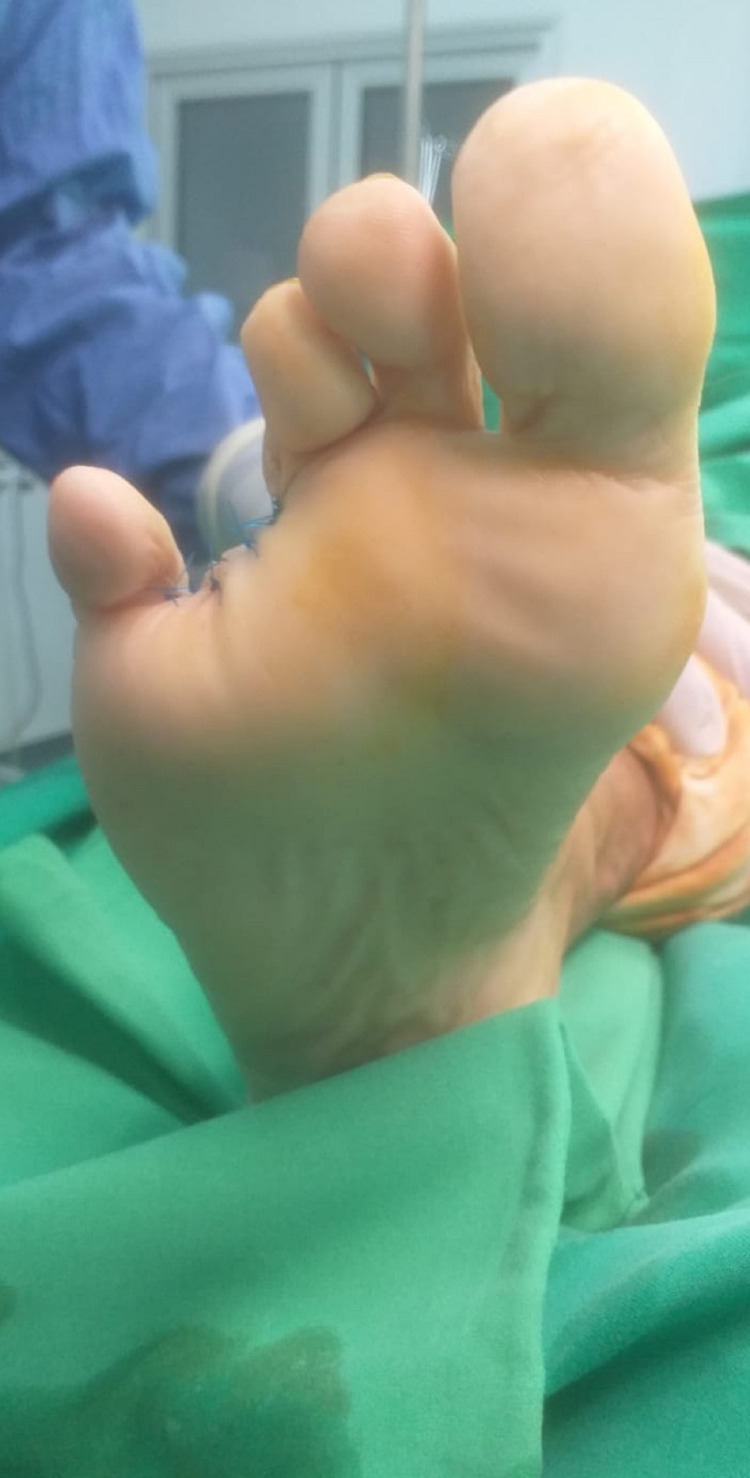
Plantar view of the foot after amputation

After realizing that his disease was advanced, the patient decided to seek palliative care only. A palliative radiation therapy for the distal phalange was not advisable because of the complete bone loss. Instead, he was referred to our orthopedic team, for a surgical intervention, aiming to amputate the affected toe. This procedure was done as a palliative intent, given the unbearable pain the patient was suffering. It was poorly controlled by opioids. The intervention was done under loco-regional anesthesia with no complications (Figures 9, 10).

**Figure 11:**
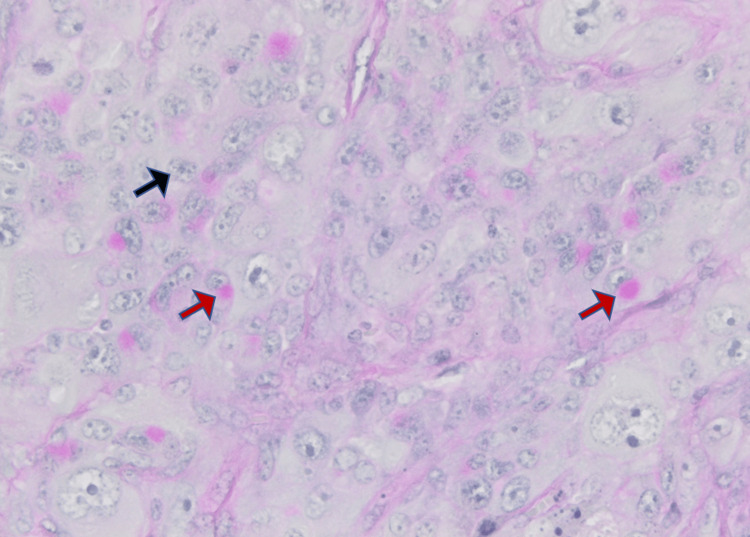
Microscopic image with periodic-acid Schiff (PAS) staining, showing a neoplastic proliferation made up of large polyclonal cells disclosing a dark and irregular nucleus with atypical mitotic figures. Focal necrosis and some vacuoles are also seen. There was no clear adenocarcinoma, squamous or neuroendocrine morphology. The black arrow shows nuclei of neoplastic cell; the red arrow shows vacuolated cytoplasm.

**Figure 12:**
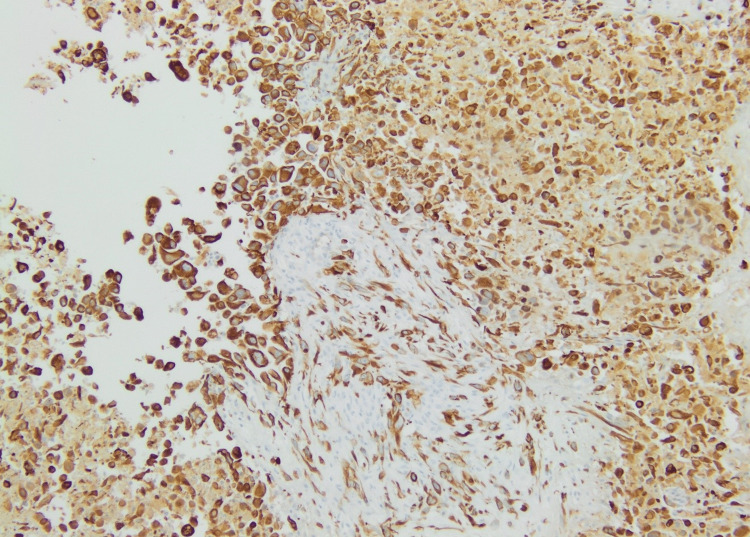
A microscopic image with immunolabelling revealing a strong expression of cytokeratin CK7 in tumor cells. TTF1 is diffusely positive in the nuclei. CK 20 and p63 are negative.

The patient was rapidly relieved from the pain a few days after his discharge from the hospital. The pathology report confirmed that the amputated toe was indeed a poorly differentiated carcinoma of pulmonary origin (Figures 11, 12).

## Discussion

A metastatic lesion to the foot is a rare entity, especially when it comes to the digits. It is known that an acrometastasis, which is a metastasis that appears below the elbow or the knee, is a very rare entity that accounts for 0.1% of bone metastases [3]. Acrometastases are most frequently found in patients having a lung cancer (especially adenocarcinoma and squamous cell carcinoma), followed by tumors in the endometrium, breast, kidney and finally in the colon-rectum [4]. The mechanism of acrometastasis is not completely defined. Many different hypotheses have been advanced, like the hematopoietic dissemination or multiple repetitive microtraumas [5]. In some cases, the acrometastasis could be the first sign of the presence of neoplasia, but they are mostly seen in patients with an established diagnosis of cancer [6]. Their presence is usually a sign of a terminal stage cancer, thus divulgating a bad prognosis with a mean life expectancy of six months [2].

The most common localization of the acrometastasis to the foot is the hindfoot (calcaneus or talus) followed by the midfoot (cuboid, navicular, cuneiforms). The presence of metastasis to the forefoot (metatarsal bone or phalanx) is the rarest [4]. The clinical presentation of phalangeal metastasis is often mistaken with an osteomyelitis or a gouty arthritis, because of the erythema, pain, and swelling [7]. Other entities can also mimic an acrometastasis, such as ganglion cysts, acute paronychial infections, pyogenic granulomas, herpes zoster, tuberculous dactylitis, erysipelas, or some skin tumors, making the diagnosis quite challenging [2,6].

Normal radiographs are the most common imaging technique used to diagnose acrometastasis [4]. Their predominance appears as lytic lesions, but this aspect depends on the primary tumor type [8]. When they originate from the lungs, they are most commonly seen as a lytic lesion to the bone, and in extremely rare cases as blastic lesions [9]. Even if the lesions can be seen on radiographs and CT scans, the gold standard imaging technique for diagnosis is the MRI, since it can reveal the extension, shape, and consistency of the tumor [6].

The main concern in the treatment of acrometastasis is the relief of pain or other symptoms, and if possible, the regain of some functional ability of the affected area [10]. In fact, many therapeutic options can be used depending on the case, such as anti-inflammatory drugs, narcotics, chemotherapy, localized radiotherapy, or even surgical interventions like curettage or amputation [11]. Amputation is proposed as a salvage procedure, after all other non-operative modalities have failed to improve pain and functional outcomes. Although it does not improve the overall prognosis, it relieves the patient from the local pain caused by the acrometastasis [11,12]. This was the case with our patient, who had a poor control of his pain, even with strong analgesics [4]. Moreover, multiple studies have shown that even if chemotherapy was used to try to relieve the pain locally and improve the primary and secondary lesions, the overall prognosis was still poor [4,13]. In the case of our patient, who was not symptomatic for all the metastasis he had, but suffered only from the intense pain of his acrometastasis, the amputation was considered as a good palliative option, compared to the high dosages of narcotics he was using to manage the pain.

## Conclusions

The case we introduced here highlights the fact that an acrometastasis is to be suspected in patients with an active large cell carcinoma of the lung, even if the tumor was initially localized to the lung parenchyma. The rapid evolution from a localized tumor to a metastatic state made the diagnosis of the acrometastasis quite challenging, as it was the first manifestation of the evolution of the cancer. The option of palliative surgery should be integrated in the therapeutic arsenal of acrometastasis, especially when the localization of the metastasis is distal and in the digits. In this case, the amputation of the toe gave the patient great pain relief, compared to the multiple side effects of the opioids or other strong analgesics needed to relieve the pain.
